# Effect of Extreme Weather Events on Mental Health: A Narrative Synthesis and Meta-Analysis for the UK

**DOI:** 10.3390/ijerph17228581

**Published:** 2020-11-19

**Authors:** Joana Cruz, Piran C. L. White, Andrew Bell, Peter A. Coventry

**Affiliations:** 1 Department of Environment and Geography, University of York, Wentworth Way, York YO10 5NG, UK; piran.white@york.ac.uk; 2 Interdisciplinary Global Development Centre, University of York, York YO10 5DD, UK; 3 Centre for Mental Health, 90 London Road, London SE1 6LN, UK; andy.bell@centreformentalhealth.org.uk; 4 Department of Health Sciences, University of York, Heslington, York YO10 5DD, UK; peter.coventry@york.ac.uk

**Keywords:** anxiety, depression, flooding, heat wave, post-traumatic stress disorder, systematic review

## Abstract

Extreme weather events are increasing in frequency and severity as a consequence of climate change and pose a significant threat to population mental health. This is the case even in temperate regions such as the United Kingdom (UK) where flooding and heat waves are forecast to become more common. We conducted a systematic review to quantify the prevalence and describe the causes of common mental health problems in populations exposed to extreme weather events in the UK. We searched Web of Science, EMBASE and PsycINFO for studies that measured the prevalence of depression, anxiety, and post-traumatic stress disorder (PTSD) in populations exposed to extreme weather events in the UK, published up to 12 December 2019. We included 17 studies, four of which were included in meta-analyses to determine the point prevalence of common mental health problems in the period within 12 months following extreme weather events. The point prevalence was 19.8% for anxiety (k = 4; *n* = 1458; 95% CI 7.42 to 32.15), 21.35% for depression (k = 4; *n* = 1458; 95% CI 9.04 to 33.65) and 30.36% for PTSD (k = 4; *n* = 1359; 95% CI 11.68 to 49.05). Key factors that affected mental ill health in people exposed to flooding were water depth and absence of flood warnings. Displacement from home underscored the narratives associated with people’s perceptions of the impact of flooding. The high prevalence of common mental health problems suggests that the prevention of mental ill health in populations at risk or exposed to extreme weather events should be a UK public health priority.

## 1. Introduction

Anthropogenic changes to Earth’s biophysical systems have already had potentially irreversible impacts on the environment that are detrimental to physical and mental health [1]. These impacts are especially visible with respect to climate change. Many of the mental health impacts of climate change are associated with extreme weather events such as tropical storms and hurricanes, heatwaves, drought and floods, which are increasing in frequency and severity worldwide [2,3], with forecasts indicating that this trend will continue for decades to come [4].

Direct exposure to extreme weather events such as hurricanes and flooding can lead to loss of life, but there are also enduring impacts on people’s mental health. These include depression, anxiety and mood disorders, post-traumatic stress, sleep disruption and suicide [5,6,7,8,9,10]. Mental health disorders are the third leading cause of years lived with disability, with a prevalence of greater than 10% across all 21 countries in the Global Burden of Disease study [11]. As well as being responsible for high levels of disability, mental health problems have a profound impact on the economy. Globally, lost productivity associated with common mental health problems such as depression and anxiety is estimated at US $1 trillion each year [12]. In the UK, mental health problems accounted for 17.5 million days lost to sickness absence in 2018, equivalent to 12.4% of all reasons for sickness absence [13]. Just considering England, in 2018, the economic and social costs of mental ill health was estimated to reach £119 billion/year [14]. Given the high personal, public health and economic burden, the need to reduce the prevalence of mental health problems is a global health priority.

There is increasing evidence that extreme weather conditions have an impact on mental health for people in the UK with those experiencing storm or flood damage to their home having poorer mental health (e.g. Ref. [15,16]). More frequent heavy winter rainfall and changes in land use are contributing to increased surface and fluvial flooding, with 1 in 6 properties now at risk [6]. Ongoing climate change means that such extreme weather-related conditions are likely to be more frequent in the UK in the future [17], and it is important to understand the potential impacts on mental health so that effective interventions to reduce these impacts can be developed.

In this paper, we present the results of a systematic review to quantify the prevalence of common mental health problems in populations exposed to extreme weather events in the UK. We have adopted a mixed-methods approach, by applying a qualitative (narrative synthesis) and a quantitative (meta-analysis) analyses, to capture findings about people’s experiences and perceptions of extreme weather events to better understand how exposure to these events drive mental ill health. The work can help inform the UK health and environment policy agenda to bring about integrated solutions to prevent and reduce mental health problems in the presence of extreme weather events.

## 2. Materials and Methods

### 2.1. Search Strategy and Selection Criteria

The systematic review was reported in accordance with the Preferred Reporting Items for Systematic Review and Meta-analysis (PRISMA) guidelines [18] (see Table S1, Supplementary Materials). The study selection followed four steps: (1) All reference records were downloaded to EndNote X9.3.1 (Clarivate Analytics, Philadelphia, USA); (2) duplicates were deleted using the software function; (3) the titles and abstracts were screened for eligibility; and (4) the potentially eligible references’ full text was evaluated. We conducted an integrated search for quantitative and mixed-methods peer-reviewed English language articles in three electronic databases: Web of Science, EMBASE and PsycINFO, from inception to 12 December 2019. The search strategy complied with PECO framework: Participants, Exposure, Comparator and Outcomes. The search strategy was a combination of setting (e.g., “England”, “United Kingdom”), exposure (e.g., “flood”, “heat wave”, “climate change”) and outcome (“mental health”, “depression”, “anxiety”, “well-being”). For this review, we used the definition of “extreme event” in Stephenson et al. [19]: “(…) events that have extreme values of certain important meteorological variables. (…) such as large amounts of precipitation (e.g., floods), high wind speeds (e.g., cyclones), high temperatures (e.g., heat waves), etc.”. The full search strategy is shown in Table S2.

Studies were screened by one reviewer (J.C.) using the eligibility criteria described below. Where eligibility was unclear, a consensus meeting with two other reviewers (P.C.L.W. and P.C.) was held to decide on inclusion. Studies were included if they met the following criteria: (1) The extreme event had taken place in the United Kingdom; (2) The study covered common mental health problems as described in the tenth edition of the International Classification of Diseases (ICD-10) [20]; (3) Mental health outcomes were measured using validated self-report scale or checklist (e.g., Generalized Anxiety Disorder scale (GAD-2); Patient Health Questionnaire (PHQ-2) depression sub-scale; short-form PTSD checklist (PCL-6)). We excluded studies that were conference abstracts, reports, reviews, meta-analyses, letters, pilot studies or protocols. Studies that only reported qualitative data and did not identify the participant population using ICD-10 diagnostic categories were also excluded.

### 2.2. Data Extraction and Quality Assessment

One reviewer (J.C.) extracted relevant data from eligible studies. The following information was extracted: authors’ names, publication year, sample size, participants’ age, gender and ethnicity, extreme climate event, location (year of event), months after the event, health outcome (e.g., anxiety, depression, post-traumatic stress disorder), health outcome assessment (e.g., Generalized Anxiety Disorder scale (GAD-2); Patient Health Questionnaire (PHQ-2) depression sub-scale; short-form PTSD checklist (PCL-6)), inclusion in meta-analysis (Y/N) and quality score (0–8) [21]. We used the eight-item Loney Criteria [21] to assess the quality of the studies. The overall score ranges from zero to eight, with each item worth one point if it met the criteria.

### 2.3. Narrative Synthesis and Meta-Analysis

Drawing on guidance for syntheses to inform policy making and research prioritization, we used a narrative synthesis approach [22]. This approach offers an efficient and practicable means to include a qualitative description and maps of findings of included studies and identify common and emergent themes about people’s experiences and perceptions about the relationship between exposure to extreme weather events and mental health. Narrative synthesis draws on the techniques of thematic analysis to categorize emergent and recurring themes within and between studies.

To quantify the point prevalence of common health problems among populations exposed to extreme weather events, we calculated an overall summary effects size by applying a fitted random effects model to each of our outcomes. To quantify the observed variability between studies, we calculated I^2^ [23], the standard deviation τ (tau) and variance of heterogeneity τ^2^ [24]. I^2^ allows us to compare estimates of heterogeneity across meta-analyses; values of 50% represent moderate heterogeneity and values above 75% represent high heterogeneity [23]. τ^2^ is the total amount of systematic differences in effects across studies [24]. All results were plotted as forest plots with 95% confidence intervals. We divided the studies into two time frames according to time after the respondent had their house flooded: ≤12 months and >12 months. Due to the reduced number of papers (1–2 studies depending on the mental morbidity), we only used in the meta-analysis and forest plots the references for ≤12 months.

We identified influencing outliers by screening for externally studentized residuals that are larger than 2 in absolute value. If one or more studies were identified as outliers, we then assessed if they were truly influential by applying a leave one out sensitivity analysis [24]. Funnel plot and Eggers’ regression test were used to evaluate potential publication bias. Statistical analyses were performed using the “meta” and “metafor” package of R version 3.6.2 (R Foundation for Statistical Computing, Vienna, Austria) [25].

## 3. Results

### 3.1. Study Selection

**Figure 1:**
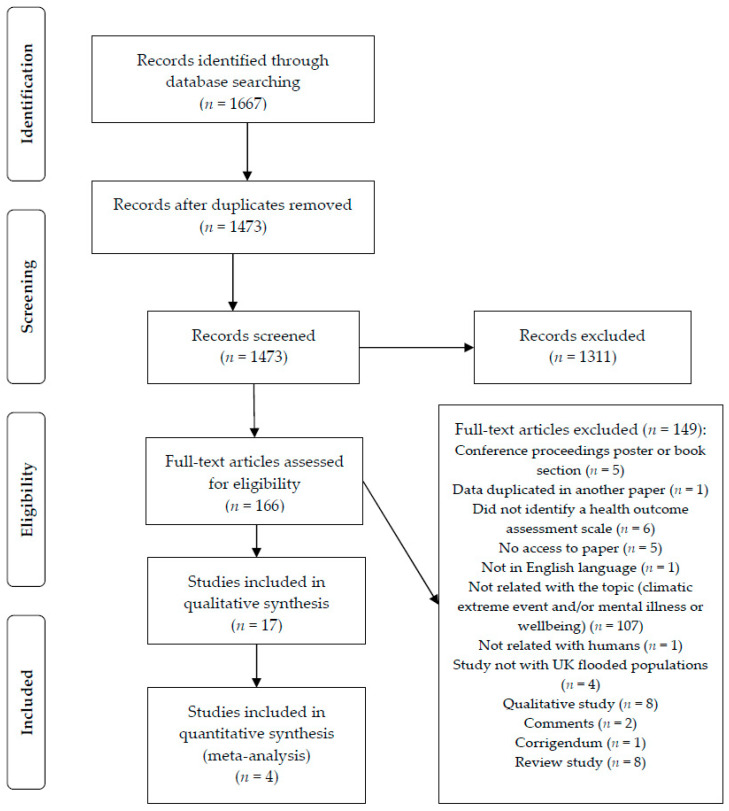
Preferred Reporting Items for Systematic Reviews and Meta-Analyses (PRISMA) flow chart of study identification process.

The database search initially identified 1667 studies, from which 194 duplicates were removed. After title and abstract screening, 1311 ineligible studies were excluded. A total of 166 potentially relevant full-text studies were independently assessed based on the selection criteria. From these, 149 studies were excluded for the following reasons: not related to extreme climatic events and/or wellbeing and mental health (*n* = 107); review studies (*n* = 8); qualitative data only (*n* = 8); conference proceedings; poster or book section (*n* = 6); no health outcome assessment scale identified (*n* = 5); no access to the paper (*n* = 5); study did not cover UK flood-affected populations (*n* = 4); not in English language (*n* = 1); data duplicated in another paper (*n* = 1); and not related to humans (*n* = 1). Seventeen studies were included in the systematic review and four studies were included in the meta-analysis (Figure 1). Only one study into heat waves was selected by applying this methodology.

### 3.2. Study Characteristics

The 17 included studies reported extreme climate weather events related to floods and heat waves with an impact on a range of mental health outcomes: anxiety, depression, post-traumatic disorder syndrome (PTSD), psychological distress and suicide ideation.

Quality scores ranged from three to seven points across studies. The minimum quality score of three was recorded for two studies and a quality score of seven was recorded for three studies (Table 1). Two studies were scored four points, five were scored five points, and five were scored six points (Table 1).

### 3.3. Narrative Analysis

Six themes were identified in the narrative and systematic review of the papers related to flooding and its impact on mental health and wellbeing: mental health morbidity; physical health and longer term effects on mental health; characteristics of the flood (e.g., increase in water depth); flood warning; displacement and loss of sense of place; and socio-economic impact. The impact of heat waves on mental health will not be analyzed in this and the following sections due to having only a single paper to report on.

#### 3.3.1. Mental Health Morbidity

The majority of studies described anxiety, depression, PTSD, suicide ideation and psychological distress as the main mental health morbidities reported by respondents as a consequence of the flood event [15,16,26,27,28,29,31,32,33,34,36]. There was no report in any of the references on the mental health condition of the respondents prior to the event.

#### 3.3.2. Physical Health and Long-Lasting Impacts on Mental Health

Alongside mental health problems, study participants also reported that they were affected by physical illnesses (e.g., earache, rash, gastroenteritis) [29,30,36,41,42]. Water quality and possible contamination of flood water with pollutants and water-borne pathogens were identified as factors that exacerbated existing psychological distress [29,30,31,36].

The long-lasting effect of flooding, from 6 to 24 months after the event, was described in several studies of flooded communities. This was illustrated by increased visits to GP practices and hospital referrals 12 months after the flood [35], and by participants self-reporting on-going psychological distress [28,29,30,33,35,36]. The risk of long-term mental health problems was reported to be between four [30] to 8.7 times [28] as high for flood victims compared with non-flooded subjects. Even years after the event, respondents affected by flooding experienced anxiety during heavy rain [29,36,41,43,44]. Anxiety was associated with increased levels of stress, sleep problems, panic attacks, difficulty concentrating on everyday tasks, lethargy, nightmares, anger, mood swings and increased use of alcohol or prescription drugs [36,41] or antidepressants [39].

#### 3.3.3. Characteristics of the Flood

Water depth in the house was associated with increased risk of psychological distress [27,29,30,31,33,34] and an increased number of attendances at GP surgeries [35]. Repeated flooding affected individuals differently: some presented increased symptoms of PTSD and anxiety [16], whilst others reported the same increased odds of psychological morbidity if exposed to a single event or repeated ones [26].

#### 3.3.4. Flood Warnings

The absence of flood warnings contributed to a significant higher score for anxiety and PTSD than a warning of 12 h or more [27,29].

#### 3.3.5. Displacement and Loss of Sense of Place

The majority of studies reported that evacuation and temporary rehousing increased the rate of psychological distress [27,31], anxiety [16,27,33], depression [16,27,33] and PTSD [16,27,29,33]. One study, covering the population of Lewes, described no impact in these mental illnesses [30]. Alongside displacement, disruption of essential services (e.g., gas, electricity and water supply) [31,33] as well as health or social services, and work or education, there were also increased odds of psychological morbidity [33]. The length of time to get the house back to normal were associated with an increased risk of psychological distress [36,42]. The odds ratio of anxiety, depression and PTSD were not related with the duration of displacement, being twice as likely for a flooded respondent to experience those conditions when they were either one or six months displaced [27]. Respondents that reported persistent flood damage to their property were more likely to suffer from depression and anxiety than flood respondents who did not suffer persistent damage [28,33].

People reported a loss of sense of place and security and the grief of losing objects that make a home [32,36,37,41,42]. Within the family context, while there were respondents who experienced a positive change in their interpersonal relations (e.g., increased bonding), others found that relationships became stressful, leading to an increase in arguments [32,37] and in some cases, to divorce [37]. At the community level, some studies reported a negative impact, with disrupted activities and loss of community spirit [32,36], while others reported increased community resilience and reduced psychological distress owing to social cohesion and collective efficacy to combat the effects of the floods [34,37,38].

#### 3.3.6. Demographic and Socio-Economic Profile of Flood Impact

The majority of studies showed that women were more likely to report psychological distress and PTSD from having their home flooded then men [15,29,31,32,35,36,37]; only one study presented similar odds of psychological distress between genders [32]. Within women respondents, ethnic minorities (Pakistani respondents) were at a higher risk of those conditions than non-Pakistani respondents [36]. Possible explanations put forward for this difference were related to women’s vulnerable social position, childcare responsibilities, usually with large families, lack of flooding experience and disbelief that these extreme events could happen in England [36]. A few studies described a different outcome: after the 1968 flood event in Bristol, there was a significant increase in male attendances to GP practices compared with before the floods [35]. A single study showed no significant difference by gender in recovery after the floods [37], while reporting that a higher percentage of women than men felt traumatized and experienced the floods as “very severe” [37]. Regarding age, respondents under 65 years reported higher levels of psychological distress than those of 65 years and over [15,29,36].

Secondary stressors affecting mental illnesses differed between men and women [32]. For women, those with concerns about pets and being separated from their family were at a high risk of self-diagnosing with anxiety and PTSD. For men, those who reported relationship problems were most likely to experience depression and anxiety [32].

Home ownership, as an indicator of income, was linked to lower levels of poor mental health when compared with those in rented accommodation [15,29]. Those with lower income levels [15,41], unemployed [31], economically inactive [15] and those with prior medical conditions [16,29,31] were more likely to experience deteriorations in their psychological health after exposure to flooding.

Other financial factors, such as problems with insurance companies or a lack of insurance, were associated with increased levels of stress immediately after flooding [29,36]. Lack of support from different authorities before, during and after the floods, which led consequently to a loss of confidence and trust, was also highlighted by the flood victims as hindering their mental recovery and increasing their levels of anxiety [36].

### 3.4. Meta-Analysis of the Prevalence of Common Mental Health Problems

All the surveys considered in the meta-analysis occurred ≤12 months after the respondents experienced their house being flooded. The included studies reported prevalence rates of between 5.9% and 27.9% for anxiety, 7.1% and 34.6% for depression and 7.06% and 43.7% for PTSD. For all three conditions, the lowest prevalence rate was reported by Graham et al. [15]

**Figure 2:**
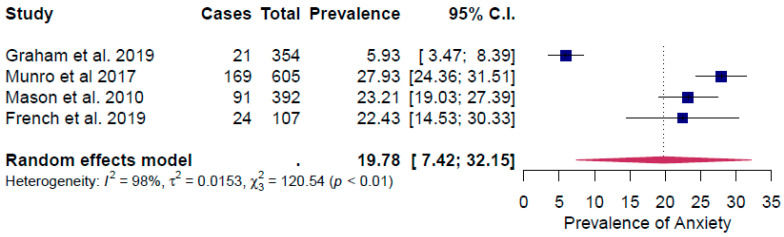
Forest plot of the prevalence rate of anxiety.

The overall point prevalence rate for anxiety associated with flooding was 19.8% (95% confidence interval (CI): 7.4–32.2%) (Figure 2). There was a high and significant heterogeneity in effect sizes between studies for anxiety: I^2^ = 98%, τ^2^ = 0.02, Q*_resid_* (3) = 120.5, *p* < 0.0001 (Figure 2).

The results from the sensitivity analysis identified the study by Graham et al. [15] as an outlier, having a significant influence on the effect size (Figure S1). By removing that study, the prevalence of anxiety was 25.2% and the effect size heterogeneity decreased significantly (I^2^ = 45%, τ^2^ = 0.0005, Q*_resid_* (2) = 3.5, *p* = 0.2).

**Figure 3:**
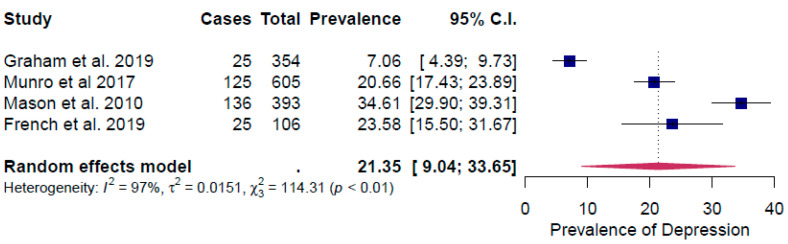
Forest plot of the prevalence rate of depression.

For depression, the aggregated value for prevalence rate for depression was 21.3% (95% confidence interval (CI): 9.0–33.7%) (Figure 3). There was a high and significant heterogeneity in effect sizes between studies for depression: I^2^ = 97%, τ^2^ = 0.02, Q*_resid_* (3) = 114.3, *p* < 0.01 (Figure 3).

The leave one out sensitivity analysis identified two studies as having a significant influence on the effect size (Figure S2)—Graham et al. [15] and Mason et al. [16]. By removing Graham et al. [15], the prevalence of depression increased to 26.3%; heterogeneity decreased but remained significant (I^2^ = 89.1%, τ^2^ = 0.005, Q*_resid_* (2) = 23.1, *p* < 0.0001) (Figure S2). Regarding Mason et al. [16], the removal of this study decreased the pooled prevalence rate to 16.7%, whilst the heterogeneity between studies remained significantly high (I^2^ = 95%, τ^2^ = 0.007, Q*_resid_* (2) = 47.2, *p* < 0.0001) (Figure S2).

**Figure 4:**
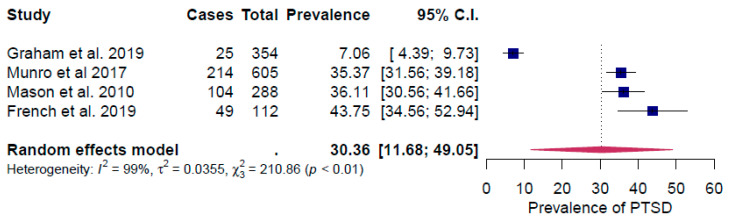
Forest plot of the prevalence rate of Post-Traumatic Syndrome Disorder (PTSD).

The point prevalence rate of PTSD was 30.4 (95% CI: 11.7–49.1) (Figure 4), which was higher than comparable prevalence rates for depression and anxiety. The heterogeneity between studies was also the highest amongst studies that measured PTSD: I^2^ = 99%, τ^2^ = 0.0355, Q*_resid_* (3) = 210.9, *p* < 0.01 (Figure 4).

In the sensitivity analysis, the study by Graham et al. [15] was again identified as an outlier, having a significant influence on the effect size (Figure S3). By removing that study, the prevalence of PTSD increased to 36% and the effect size heterogeneity decreased significantly (I^2^ = 0.01%, τ^2^ = 0.000, Q*_resid_* (2) = 2.7, *p* = 0.3).

Beggs’ tests of funnel asymmetry for anxiety, depression and PTSD were non-significant (*p* > 0.05), which indicated no publication bias.

## 4. Discussion

The point prevalence of common mental health problems was high in populations exposed to floods in the UK. The impact of the floods led to short-term and long-term mental health problems and contributed to additional problems such as the use of alcohol and prescription drugs and increased healthcare resource use among those affected. Critical features of the floods associated with mental health problems were water depth and absence of flood warning. Secondary stressors that characterized responses to flooding included evacuation and displacement and disruption to services and amenities and an absence of post-flood support. There was modest evidence of heightened resilience and no greater risk of mental health problems in populations exposed to repeat flooding compared with single flooding. Social and economic inequalities were also evident. Women experienced a greater mental health impact than men, as did people who rent rather than own their home, and in the one study that included ethnicity, there was a greater risk among those from a minority ethnic community.

The observed prevalence rate of PTSD among populations exposed to flooding in the studies included in this review (30.4%) is substantially higher than the lifetime prevalence rate of 7.8% observed in the general population [43] (Table 2). The lifetime prevalence rate for major depressive disorder in the general population has increased over the past 25 years and is estimated to be 20.6%, which is comparable with the prevalence rate for depression identified in this review (21.4%) [44] (Table 2). Generalized anxiety disorder is a relatively common disorder with a 12-month prevalence of 3.1% and a lifetime prevalence of 5.7%. We found a very high prevalence rate for anxiety (19.8%) in the included studies, suggesting that reports of anxiety also overlapped with reports of anxiety disorders as a whole, which have a lifetime prevalence of 28.8% [45] (Table 2). As reported in the Results section, the study by Graham et al. [15] was an outlier to the other studies regarding the impact of flooding on the prevalence of anxiety, depression and PTSD. Graham et al. [15] based their analysis on a stratified sample of the UK population, that was not specifically related to a flooding event. In contrast, the other three studies reported in Figure 2, Figure 3 and Figure 4 were conducted in geographical areas where severe flooding had occurred or with groups of individuals who had been exposed directly to flooding. In the Graham et al. [15] study, severe flood damage (having to leave the home for at least three days, or being unable to leave the home for at least three days) was recorded by only seven out of 7525 participants. The equivalent figures are 449 of 2126 respondents for the Munro *et al.* [27] study and 269 or 444 responses for the Mason et al. [16] study. An additional reason for the difference observed is likely to be because the Graham et al. [15] study related to “storm and flood-related damage”, and not only flood damage. Based on insurance claims data, floods represent 19% of the overall storm- and flood-related damage [15]. Although the costs of flood-related claims are > 3.5 times higher than other storm damage [15] and may be expected to lead to higher mental health impacts relative to other storm damage, the overall prevalence of anxiety, depression and PTSD reported by Graham et al. [15] would therefore be expected to be lower.

When compared with international evidence about the impact of flooding, we see that the prevalence rates identified in our review compare with those reported for depression (17.2%) and PTSD (22.4%) in the aftermath of floods in South Korea [46]. Much lower prevalence rates for PTSD were reported in a retrospective study of flooding in Hunan, China, although in keeping with our findings, this study reported that flood severity and female sex were associated with higher odds of PTSD [47].

Significantly, the level of mental health problems observed among flood victims compares with those observed among displaced populations in conflict zones [48]. Displacement and loss of a sense of place and home were major themes that underscored reports of common mental health problems among flooded populations. This was true even one year after flooding, which suggests that displacement is an important secondary stressor that drives longer term mental health outcomes after flooding. The loss of a sense of place that stemmed from displacement relates to the concept of place attachment, which is a concept that describes the psychological and emotional bonds between people and places [49]. Disruption to these bonds can lead to solastalgia, which refers to distress caused by environmental degradation and loss of home and belongings. Moreover, as reported in the narrative synthesis, a breakdown in social ties and community spirit were also associated with displacement and disruption to a sense of place, highlighting the importance of social capital for the maintenance of well-being [38].

### 4.1. Implications for Research and Policy

Improving the early identification of mental health problems and increasing access to treatment is a global public health priority in the context of natural disasters such as flooding. While there are existing evidence-based psychological therapies recommended for managing depression and anxiety, such approaches are typically only made available to clinical populations with above-threshold symptoms. In the context of flooded populations, there is a need to consider how best to provide appropriate and effective psychological care at scale for people with a broader range of mental health symptoms. Furthermore, there is a case to consider preventive approaches that build resilience in communities at risk of flooding. There is no strong evidence that psychological de-briefing is effective in disaster contexts and this approach is not warranted [50]. However there is a growing understanding that help-seeking for mental health might be encouraged by greater community mental health literacy and the influence of social networks [51]. Mental health literacy refers to knowledge and beliefs about mental health which can aid the recognition, management and prevention of mental health difficulties. In view of this, mental health first aid is designed to increase knowledge about common mental health problems and reduce stigma and provide the means to offer immediate help and signpost to professional services. There is modest evidence that mental health first aid is associated with small to medium effects for improving knowledge about attitudes towards mental health and to promote help-seeking behavior [52]. Furthermore, there is emerging evidence that mental health first aid can improve mental health literacy and facilitate appropriate support for people with mental health problems [53], suggesting that populations at risk from flooding would benefit from the roll out of such programmes. However, formal evaluation of mental health first aid is scarce and more robust assessments are needed to help make evidence-based decisions about its potential effectiveness and implementation [54].

More broadly, there is scant evidence about the effectiveness of acute psychological interventions for disaster-related mental health problems. There are calls within psychiatry to heed a warning about reducing trauma to a formula about exposure and treatment and instead see disaster-related trauma as intertwined within the more complex context of pre-existing and comorbid mental health problems, including exposure to previous traumatic events [55]. This calls for novel approaches that draw on systems thinking that can propose integrated solutions to address the connections between climate change and climate mitigation and the persistent social determinants of mental ill health [56]. Here the role of interventions that can enhance community and environmental resilience, including improved risk communication [57] and explicitly incorporating flood risk and management into urban planning [58] could be key to addressing the mental health challenges posed by climate change in the 21st century.

The findings have implications for local authorities and national governments alike. They imply that steps to reduce the risk of climate-related disasters will have long-term benefits to the public purse and society more widely by reducing the risk of long-term mental ill health in communities. Mental ill health carries a heavy cost for individuals, families and communities, and actions to prevent it can present good value for money. As well as taking action to reduce the risk of climate-related disasters, local authorities can also take steps to build community resources and resilience, especially among the most marginalized and disadvantaged social groups [59].

The findings also have significance for resource allocation and planning of mental health services. Flooding, and potentially other climate-related disasters, increase a community’s risk of mental ill health. Localities that have experienced or face a high risk of flooding may need more investment in psychological support, and this will be needed in the long-term, and not just in the form of a crisis response.

This paper also identifies some major gaps in research that need to be addressed if we are to build resilience against the psychological impacts of climate disasters. While we were able to draw conclusions about the impacts of flooding, there is insufficient evidence relating to other types of incidents, including heatwaves. Additionally, we found that people from more deprived backgrounds who rented property had poorer psychological health after flooding. Future qualitative research could address questions about the differential impact of flooding among populations in relation to income, home ownership and ethnicity.

### 4.2. Strengths and Limitations

A strength of this review is that we included studies that included reports of common mental health problems and not just PTSD, making it a comprehensive assessment of the prevalence of mental ill health in flood-exposed populations in the UK. Furthermore, we only included studies that identified populations with identifiable mental health problems that map to valid diagnoses, thereby enabling a comparison of our findings with wider epidemiological evidence about mental health in the general population. Our review also included studies on the impact of fluvial flooding, which is the most common form of flooding in the UK, making it more policy-relevant for the UK and other contexts where this form of flooding poses the greatest threat. By including a qualitative narrative synthesis, we were also able to highlight contextual factors that underpinned the onset and maintenance of mental health problems among flood victims.

Our review has a number of limitations. First, while our review offers a comprehensive assessment of the impact of flooding on mental health, our search only identified a single study that addressed the mental health impact of other climate change extreme weather events (heat waves). However, by focusing on the impact of fluvial flooding and heat waves, our review provides a strong signal for further research about the mitigation of the impact of extreme weather events most likely to affect the UK. Secondly, we included in the meta-analysis studies that reported both short-term (<6 months) and longer-term (between 6 and 12 months) mental health outcomes and we were unable to differentiate between the direct and indirect effects of flooding on mental health. As indicated by the qualitative synthesis, the role of secondary stressors is especially important in understanding how interventions might mitigate and manage the longer-term mental health impact on communities affected by floods. Thirdly, most included studies were uncontrolled, and it was not possible to ascertain the comparative prevalence of mental health problems in unexposed populations. Finally, the included studies displayed high levels of heterogeneity in terms of type of flood and flood severity and meta-analysis of these studies precluded the means to disentangle the impact of these factors on the prevalence of mental health problems.

## 5. Conclusions

This systematic review showed that populations exposed to floods have a high prevalence of common mental health problems, with the rate for PTSD and anxiety disorders exceeding lifetime prevalence rates in the general population. Mental health problems were especially attributable to displacement and loss of sense of place and home and disturbances to social capital, and they affect those with the least resources disproportionately. Climate change has significantly increased the prospect of more frequent extreme weather events and there is an urgency to develop solutions to support people’s short- and long-term mental health following events such as floods. There is scope for more research that can address how to mitigate the risk associated with extreme weather events and build resilience within communities affected by floods.

## Figures and Tables

**Table 1:** Characteristics of the studies included in the narrative review and meta-analysis of the impact of floods and heat waves in mental health outcomes and wellbeing.

Study	Event	Location (Year of Event)	Months after the Event	Respondents’ Characteristics	Health Outcome	Health Outcome Measurement	Included in Meta-Analysis (Y/N)	Quality Score (0–8)
French et al. [26]	Flood	Cumbria (2015/16)	6	Flooded: 119; Gender: 59%; Ethnicity: 100% white; Marital status: 64% married/civil partners or cohabiting; Housing tenure: 82% owned house; Employment: 52% employed and 40% retired; Education level: 45% with degree or above, 32% below degree; English deprivation quintile: 1.7% in least deprived quintile; 6% in quintile 4 and 5 (most deprived); Long-term illness: 72% yes	Depression; Anxiety; Post-traumatic stress disorder (PTSD); Health-related quality of life	Patient Health Questionnaire (PHQ-2) depression sub-scale; Generalized Anxiety Disorder scale (GAD-2); short-form PTSD checklist (PCL-6); 5 level EQ-5D (EQ-5D-5L)	Y	5
Graham et al. [15]	Flood	England (2013/2014)	6	Flooded: 354; Age: 26% aged 16–34, 39% aged 35–54; 27% aged 55–74 and 8% 75+; Ethnicity: 89% white, 1% black, 7% Asian; Education level: 31% with degree, 14% teaching, HND and nursing, 14% A level, 26% GCSE or equivalent, 13% no qualifications; Housing tenure: 76% owned house; Employment: 69% employed, 28% economically inactive and 3% unemployed; English deprivation quintile: 29% in least deprived quintile, 34% in quintile 4 and 5 (most deprived)	Depression; Anxiety; Obsessive compulsive disorder; Panic disorder; Phobias; PTSD; Suicide ideation	Clinical Interview Schedule–Revised (CIS–R); PTSD Checklist Civilian Version (PCL–C)	Y	7
Mason et al. [16]	Flood	Anonymized	6	Gender: 182 males and 262 females; Mean Age: 57 years (SD = ±15 years); Employment: 46% employed, 0.9% unemployed and 33.6% retired	Depression; Anxiety; PTSD;	Hopkins Symptoms Checklist; Harvard Trauma Questionnaire	Y	6
Munro et al. [27]	Flood	Counties of Gloucestershire, Wiltshire, Surrey, Somerset, and Kent (2013/2014)	12	Flooded: 605 Age: 6.3% aged 18–35, 54% 36–64; 28.8% aged 65–79 and 8% 80+; Marital status: 69.3% married/civil partners or cohabiting; Housing tenure: 90.4% owned house; Employment: 49.8% employed and 3.8% retired; Education level: 37.0% with degree or above, 39.2% below degree; English deprivation quintile: 26.2% in least deprived quintile; 2.6% in quintile 4 and 5 (most deprived); Long-term illness: 22% yes	Depression; Anxiety; PTSD	Patient Health Questionnaire (PHQ-2) depression sub-scale; Generalized Anxiety Disorder scale (GAD-2); short-form PTSD checklist (PCL-6)	Y	6
Jermacane et al. [28]	Flood	England (2013/2014)	24	Flooded: 339	Anxiety; Depression; PTSD	Patient Health Questionnaire (PHQ-2) depression sub-scale; Generalized Anxiety Disorder scale (GAD-2); short-form PTSD checklist (PCL-6)	N	6
Tunstall et al. [29]	Flood	England and Wales (1998)	60	Flooded: 982 respondents	Anxiety; Depression; PTSD; Psychological distress; Suicide ideation	General Health Questionnaire (GHQ-12); Post-Traumatic Stress Scale (PTSS)	N	5
Reacher et al. [30]	Flood	Lewes (2000)	9	Flooded: 227; Gender: 123 females; Age: 22% aged 0–17, 24% 18–39; 17% aged 40–49, 22% aged 50–64 and 15% 65+	Psychological distress	General Health Questionnaire (GHQ-12)	N	7
Paranjothy et al. [31]	Flood	South Yorkshire and Worcestershire (2007)	South Yorkshire: 3 Worcestershire: 6	Gender: 72% females in South Yorkshire and 57% females in Worcestershire; Mean age: 50 years (SD = ±17 years) in South Yorkshire and 57 years (SD = ±17 years) in Worcestershire; Employment: 28% unemployed and 24% retired in South Yorkshire; and 39% unemployed and 9% retired in Worcestershire	Depression; Anxiety; PTSD; Psychological distress	Patient Health Questionnaire (PHQ-9) depression sub-scale; Generalized Anxiety Disorder scale (GAD-7); short-form PTSD checklist (PCL-6); General Health Questionnaire (GHQ-12)	N	6
Tempest et al. [32]	Flood	Anonymized (2013/2014)	12	Flooded: 622	Depression; Anxiety; PTSD	Patient Health Questionnaire (PHQ-2) depression sub-scale; Generalized Anxiety Disorder scale (GAD-2); short-form PTSD checklist (PCL-6);	N	6
Waite et al. [33]	Flood	Counties of Gloucestershire, Wiltshire, Surrey, Sedgemoor, South Somerset, and Tonbridge and Malling (2013/2014)	12	Collected but not provided	Depression; Anxiety; PTSD	Patient Health Questionnaire (PHQ-2) depression sub-scale; Generalized Anxiety Disorder scale (GAD-2); short-form PTSD checklist (PCL-6);	N	5
Greene et al. [34]	Flood	South Yorkshire and Worcestershire (2007)	1–7	2029 responders (flooded and unaffected); Mean Age: South Yorkshire: 50 years (SD = ±17 years), Worcestershire: 57 years (SD = ±17 years)	Psychological distress	General Health Questionnaire (GHQ-12)	N	5
Bennet [35]	Flood	Bristol (1968)	12	Flooded: 88 males and 109 females	Psychiatric complaints	Self-reported	N	3
Tapsell and Tunstall [36]	Flood	Banbury and Kidlington (1998)	7; 12; 54	Gender: 11 males and 21 females	Anxiety; Depression; Suicide ideation; Psychological distress	General Health Questionnaire (GHQ-12)	N	4
Akerkar and Fordham [37]	Flood	Tewkesbury (2007) Morpeth (2008)	Tewkesbury: 18 Morpeth: 12	Gender: Tewkesbury: 60 males and 76 females; Morpeth: 90 males and 146 females	Wellbeing	Mental Health Inventory (MHI-5); SF-12 Patient Questionnaire (SF-12)	N	3
Wind and Komproe [38]	Flood	Morpeth (2008)	12	Flooded: 231; Gender: 61% females; Age: 2.7% aged 18–24, 9% aged 25–39, 42.6% aged 40–64 and 57.4% 65+; Marital status: 38.4% married/civil partners or cohabiting; Housing tenure: 90.4% owned house; Employment: 32.3% employed and 57.3% retired; Education level: 22% with degree or above	PTSD	PTSD Checklist Civilian Version (PCL-C)	N	5
Milojevic et al. [39]	Flood	England (2011/2014)	NA	NA	Depression	Number of antidepressants prescribed	N	4
Page et al. [40]	Heat wave	England and Wales (1995 and 2003)			Suicide	Suicide counts	N	7

**Table 2:** Mental health morbidity prevalence for population exposed to flooding and general population for anxiety, depression and post-traumatic stress disorder (PTSD).

Mental Health Morbidity	Prevalence in Population Exposed to Flooding (%)	Prevalence in General Population (%)
Anxiety	19.8	5.7
Depression	21.4	20.6
PTSD	30.4	7.8

## Supplementary Materials

The following are available online at https://www.mdpi.com/1660-4601/17/22/8581/s1, Table S1: PRISMA 2009 checklist, Table S2: Full search strategy, Figure S1: Forest plot of the leave one out sensitivity analysis for anxiety, Figure S2: Forest plot of the leave one out sensitivity analysis for depression, Figure S3: Forest plot of the leave one out sensitivity analysis for Post-Traumatic Syndrome Disorder (PTSD).
