# Supplementary material for: Effect of Extreme Weather Events on Mental Health: A Narrative Synthesis and Meta-Analysis for the UK
Source: Int J Environ Res Public Health. 2020 Nov 19;17(22):8581. doi: 10.3390/ijerph17228581 (PMC7699288; doi:10.3390/ijerph17228581)
Supplement: Supplementary file 1 [file ijerph-17-08581-s001.zip › Table S2 - Full search strategy.docx]

Table S2 – Full search strategy: a) PECO (Population, Exposure, comparator and outcome) and b) Web of Science and c) PsycINFO and Embase string of words

| **Population** | **Exposure** | **Comparator** | **Outcome** |
| --- | --- | --- | --- |
| UK  England  Wales  Scotland  Northern Ireland  Britain  United Kingdom | Climate change  Natural disaster  Extreme events  Drought  Flood  Heat waves  Extreme heat  temperature  Extreme climatic  Wildfire  Hurricane  Cyclone  Storm  Fire and peat  Fire and forest  Fire and moor*  Air pollution  Ozone  Particulate matter  PM2.5  PM10 | N/A | Mental Health  Mental illness  Wellbeing  Well-being  Depression  Schizophrenia  Bipolar affective disorder  Psycho affective disorder  Psychoses  Posttraumatic stress disorder  PTSD  Morbidity  Anxiety  Suicide  personality disorder  trauma  psychological health  emotional health |

b) Web of Science

TS = ((UK OR England OR Wales OR Scotland OR "Northern Ireland" OR "United Kingdom" OR Britain) AND

("Climate change" or "Natural disaster" OR "Extreme events" OR Drought OR Flood* OR "Heat waves" OR heatwaves OR "Extreme heat" OR temperature OR "Extreme climatic" OR Wildfire OR Hurricane OR Cyclone OR Storm OR “forest fire” OR (Fire AND (peat OR moor*)) OR "Air pollution" OR Ozone "Particulate matter" OR "PM2.5" OR "PM10") AND

("Mental Health" OR "Mental illness" OR wellbeing OR "well-being" OR Depression OR Schizophrenia OR "Bipolar affective disorder" OR bipolar OR "psycho affective disorder" OR "psychoaffective disorder" OR Psychos* OR "Posttraumatic stress" OR "Post-traumatic stress" OR "PTSD" OR morbidity or anxiety OR suicid* OR "personality disorder" OR "trauma" OR "psychological health" OR "emotional health" ))

NOT TS = ("New South Wales" OR "New England")

c) PsycINFO and Embase

UK OR England OR Wales OR Scotland OR ‘Northern Ireland’ OR ‘United Kingdom’ OR Britain) AND

‘Climate change’ or ‘Natural disaster’ OR ‘Extreme events’ OR Drought OR Flood* OR ‘Heat waves’ OR heatwaves OR ‘Extreme heat’ OR temperature OR ‘Extreme climatic’ OR Wildfire OR Hurricane OR Cyclone OR Storm OR Fire "Air pollution" OR Ozone "Particulate matter" OR "PM2.5" OR "PM10" AND

(‘Mental Health’ OR ‘Mental illness’ OR wellbeing OR ‘well-being’ OR Depression OR Schizophrenia OR ‘Bipolar affective disorder’ OR bipolar OR ‘psycho affective disorder’ OR ‘psychoaffective disorder’ OR Psychos* OR ‘Posttraumatic stress’ OR ‘Post-traumatic stress’ OR ‘PTSD’ OR morbidity or anxiety OR suicid* OR ‘personality disorder’ OR ‘trauma’ OR ‘psychological health’ OR ‘emotional health’))
