# Supplementary material for: Effect of Extreme Weather Events on Mental Health: A Narrative Synthesis and Meta-Analysis for the UK
Source: Int J Environ Res Public Health. 2020 Nov 19;17(22):8581. doi: 10.3390/ijerph17228581 (PMC7699288; doi:10.3390/ijerph17228581)
Supplement: Supplementary file 1 [file ijerph-17-08581-s001.zip › Figures S1-S3_Florest Plot of the leave one out sensituvity analysis.docx]

Figures S1 to S3: Florest Plot of the leave one out sensitivity analysis for the three mental illnesses outcomes: anxiety, depression and PTSD


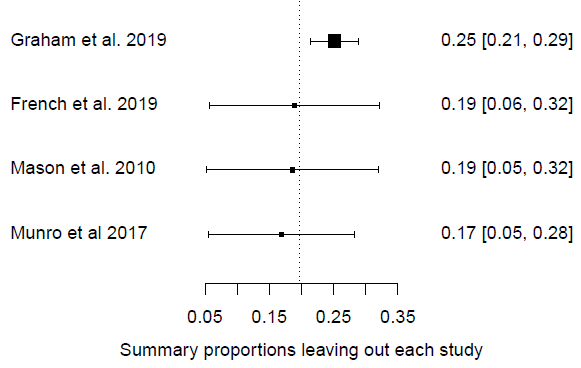


**Figure S1.** Forest plot of the leave one out sensitivity analysis for anxiety prevalence.


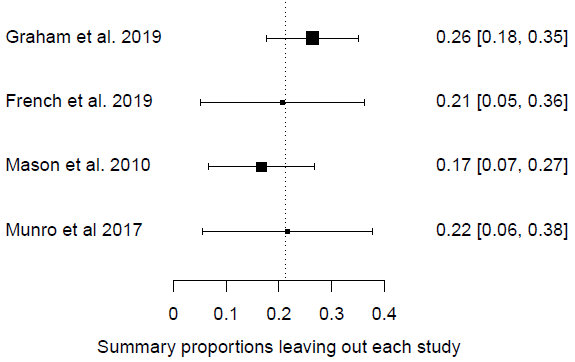


**Figure S2.** Forest plot of the leave one out sensitivity analysis for depression prevalence.


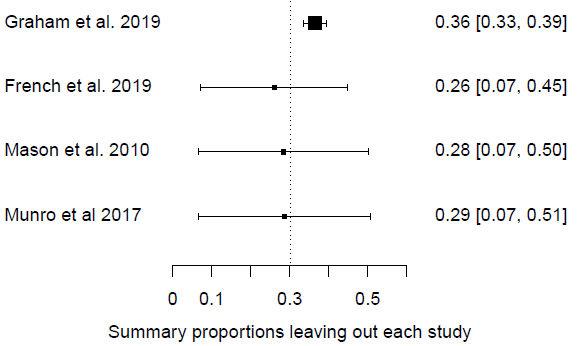


**Figure S3.** Forest plot of the leave one out sensitivity analysis for Post-Traumatic Syndrome Disorder (PTSD) prevalence.
